# Interleukin-28B rs12979860 C/T Polymorphism and Acute Cellular Rejection after Liver Transplantation

**Published:** 2017-02-01

**Authors:** H. Fereidooni, N. Azarpira, R. Yaghobi, A. Vahdati, S. A. Malek-Hoseini

**Affiliations:** 1 *Department of Physiology, Fars Science and Research Branch, Islamic Azad University, Shiraz, Iran*; 2 *Transplant Research Center, Stem Cell Research Institute, Shiraz University of Medical Sciences, Shiraz, Iran *; 3 *Department of Physiology, Shiraz Branch, Islamic Azad University, Shiraz, Iran*; 4 *Transplant Center, Shiraz University of Medical Sciences, Shiraz, Iran*

**Keywords:** Liver transplantation, Rejection, Interleukin, IL-28B polymorphism, Cirrhosis

## Abstract

**Background:**

Interleukin-28 (IL-28B) rs12979860 C/T polymorphism is a known predictor of sustained virological response after antiviral treatment in hepatitis C. IL-28B affects the innate immune system as well as intrahepatic expression level of interferon-stimulated genes.

**Objective:**

To investigate the effect of recipient IL-28B polymorphism on occurrence of acute rejection after liver transplantation.

**Methods:**

140 liver allograft recipients were selected. Acute rejection episodes were recorded in 39 patients (AR group); the remaining had normal graft function (non-AR group). 70 normal subjects were also studied as the control group. The IL-28B rs12979860 was genotyped through PCR-RFLP method.

**Results:**

No significant difference was found between AR and non-AR groups in terms of genotype and allele frequency. However, the CC genotype was significantly (p<0.001) more frequent in patients than in the control group; the **C** allele variants increased the risk of end-stage liver disease (OR: 2.60).

**Conclusion:**

Liver damage in association with the carriage of IL-28B C allele is associated with a higher likelihood of developing cirrhosis.

## INTRODUCTION

Liver transplantation (LT) is the optimal therapy for patients with end-stage liver disease. The liver is an immune tolerant organ, but the occurrence of acute cellular rejection (ACR) after LT has been recorded from 30% to 70% in the first year. Recurrent episodes of ACR may lead to long-term organ damage [1].

ACR is effectively controlled by the use of immunosuppressive drugs, especially calcineurin inhibitors (CNI). Unfortunately, administration of CNIs is accompanied with different side effects such as renal failure, diabetes mellitus, hypertension, and hyperlipidemia [2]. 

The inflammatory environment in which the recipient immune system reacts with donor antigens plays a crucial role for successful organ engraftment [3]. The genes coding for molecules responsible for the immune response such as human leukocyte antigen (HLA), interleukin-6 (IL-6) [4], interferon (IFN)-F [4], cytotoxic T-lymphocyte antigen 4 [5], and HLA-G [6], may affect the level of different cytokines with consequent impact on the balance between rejection and tolerance [3].

IL28B gene encodes interferon-λ3, triggers the activation of innate immune response with powerful antiviral activity, especially in spontaneous clearance of hepatitis C virus (HCV) [7, 8]. Recent genome-wide association studies (GWAS) have demonstrated the impact of favorable CC genotypes of IL28B rs12979860 polymorphism on the treatment response for HCV, as well as spontaneous HCV clearance in HIV coinfected individuals [10-15]. The association between IL28B rs12979860 polymorphism and ACR after orthotopic LT is also mentioned [16]. The objective of this study was to investigate whether recipient IL-28B rs12979860 C/T polymorphism may have a role in ACR after LT.

## MATERIALS AND METHODS

A total of 140 liver allograft recipients was selected during 2013 to 2015. The patients were followed for at least six months. Among these patients, 39 experienced an episode of acute rejection (AR group); the remaining (n=101) had normal graft function (non-AR group). An acute rejection episode was defined based on biopsy findings according to Banff criteria [17]. 

The Ethics Committee of Shiraz University of Medical Sciences approved the study protocol. Informed written consents were taken from all the participants. The patients received a treatment protocol consisting of tacrolimus (Prograf), mycophenolate mofetil, and steroids. Tacrolimus was started at 0.1 mg/kg/day; the dose was then adjusted to keep trough levels between 10 and 12 ng/mL in the first month post-transplantation and subsequently between 8 and 10 ng/mL. Additionally, mycophenolate mofetil was given with an initial oral dose of 2.0 g/day. Methylprednisolone was given 1 g on the day of surgery, and prednisolone 20 mg/day tapered down to zero during the first three months. Seventy normal subjects with no past history of liver disease were also included in the study as the control group. 

Genomic DNA was extracted from buffy coats using a DNA purification DNPTM kit (Cinagene, Iran). The IL-28B rs12979860 was genotyped through PCR-RFLP method. The PCR products were then digested with Hpy166II (Biolab, New England) at 37 ^o^C for 16 hrs and were observed using electrophoreses and ethidium bromide. The primer sequences, PCR condition and product size after digestion are presented in Table 1. One product from each genotype was sequenced with ABI sequence Genetic Analyzer (Applied Biosystems, Foster City, CA) in order to confirm the results.

**Table 1:** The primer sequences, and PCR condition used for genotyping of IL28B (rs12979860)

PCR primers	PCR protocol	Fragment size (bp) after enzyme digestion
5’-CGCCAGGGCCCCTAACCTCT-3’ 5’-CCCAGCAGGCGCCTCTCCTA-3’	Denaturation: 94 °C; 5 min, 94 °C; 45 sec, 62.4 °C; 1 min, 72 °C; 1 min (×35 cycles)	CC: 220TT: 190CT: 220+190

Statistical Analysis

The categorical and quantitative variables were compared with χ^2^ test and *Student’s t* test, respectively. Allele and genotype frequencies were calculated in the studied liver transplant recipients by direct gene counting. The statistical analyses were performed with SPSS^®^ for Windows^®^ ver 16 (SPSS Inc, Chicago, IL, USA). A p value <0.05 was considered statistically significant.

## RESULTS

Comparison of the AR and non-AR groups is presented in Table 2. Among 140 consecutive recipients, 62.9% were male and 37.1% were female. The mean±SD age of the patients in AR and non-AR groups was 37.7±2.4 and 36.6±1.8 years, respectively. Male-to-female ratio (M/F) was 1.4 (23/16) in AR group and 1.3 (65/36) in non-AR recipients.

**Table 2:** Demographic data of patients enrolled in the study

	AR Group (n=39)	Non-AR Group (n=101)	p value
Recipient Sex			
Males	23 (59%)	65 (64.6%)	0.55
Females	16 (41%)	36 (35.6%)	
Recipient age	37.0±2.4	36.5±1.8	0.94
Donor Sex			
Males	18 (46%)	29 (28.7%)	0.27
Females	4 (10%)	13 (12.9%)	
Donor age	36.9±3.0	27.1±2.6	0.85
Blood group			
A	17 (44%)	28 (27.8%)	0.711
B	6 (15%)	14 (13.9%)	
AB	2 (5%)	7 (6.9%)	
O	14 (36%)	24 (23.8%)	
MELD score	20.5±0.8	21.9±1.0	0.94
Banff grade			
Mild	31 (79%)		
Moderate	6 (16%)		
Severe	2 (5%)		
Immunosuppressive drugs			
Tacrolimus dose	4.25±2.43	2.34±1.24	0.08
Cellcept dose	2.88±1.14	1.17±1.56	0.09

The two groups were not significantly different in terms of donor’s age, donor’s sex, recipient’s age, recipient’s sex, MELD score, and blood group. No significant difference was also found in terms of tacrolimus and cellcept daily dosage. Furthermore, autoimmune hepatitis was more prevalent in the AR group (Table 3). HCV was the underlying cause of cirrhosis for one patient in AR group and two recipients in non-AR group.

**Figure 1:**
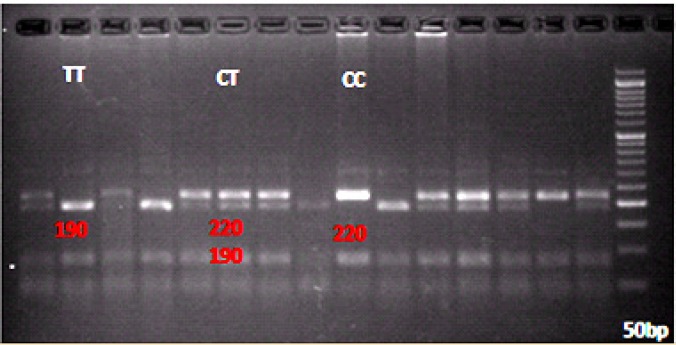
Different genotypes of IL28B (rs12979860) after enzyme digestion.

The distribution of IL-28B rs12979860 genotype and allele is presented in Tables 4 and 5. The allele frequency followed the Hardy-Weinberg equilibrium state. Direct sequencing confirmed the PCR-RFLP results (Fig. 1).

**Table 3:** Prevalence of the underlying diseases in patients with rejected liver transplants

Underlying disease	AR group (n=39)	Non-AR Group (n=101)	p value
Cryptogenic	10 (26%)	26 (25.7%)	0.99
Hepatitis B and C	9 (23%)	24 (23.8%)	0.93
Autoimmune hepatitis	6 (15%)	8 (7.9%)	0.19
PBS, PSC	6 (15%)	18 (17.8%)	0.73
Congenital diseases	8 (21%)	25 (24.8%)	0.59

PBS: Primary biliary cirrhosis; PSC: Primary sclerosing cholangitis

Congenital disease: Tyrosinemia, hypercholesteremia, Wilson

**Table 4:** Genotype and allele frequency of IL-28B (rs12979860) polymorphism in liver transplant recipients

Genotype	AR (n=39)	Non-AR (n=101)	OR (95% CI)
CC	31 (80%)	82 (81.2%)	0.9 (0.33–2.5)
CT	6 (15%)	10 (9.9%)	1.65 (0.49–5.48)
TT	2 (5%)	9 (8.9%)	0.55 (0.08–2.95)
CC+CT	37 (95%)	92 (91.1%)	0.55 (0.08–2.95)
TT	2 (5%)	9 (8.9%)	
C	68 (87%)	174 (86.1%)	1.09 (0.48–2.56)
T	10 (13%)	28 (13.9%)	

(CT: 220+190 bp, CC: 220 bp, TT: 190 bp)

No significant difference was found between AR and non-AR groups in terms of genotype and allele frequency (Table 4). There was no significant correlation between IL-28 genotypes and Banff grades. However, the CC genotype and C allele of rs12979860 was significantly more frequent in patients compared to the control group (p<0.001). Presence of a C allele variant was associated with a higher risk of cirrhosis (OR 2.60) (Table 5).

**Table 5:** Genotype and allele frequency of IL-28B (rs12979860) polymorphism in liver transplant recipients and normal controls

Genotype	Recipients (n=140)	Controls (n=70)	OR (95% CI)
CC	110 (78.6%)	32 (46%)	4.35 (2.24–8.5)
CT	18 (12.9%)	32 (46%)	0.18 (0.8–0.37)
TT	12 (8.6%)	6 (9%)	1.0 (0.33–3.15)
CC+CT	128 (91.5%)	64 (91%)	1.0 (0.32–3.04)
TT	12 (8.6%)	6 (9%)	
C	238 (84.6%)	47 (67%)	2.60 (1.56–4.43)
T	42 (15.4%)	22 (31%)	

## DISCUSSION

Genome-wide association technology recently confirmed that in patients chronically infected with HCV, the IL28B polymorphisms (rs8099917 and rs12979860) are strongly associated with responses to pegylated interferon (PEG-IFN) and ribavirin (RBV) therapy [12]. The important role of IL28B genotype with spontaneous clearance of HCV is also documented [18, 19].

IL28B gene encodes IFN-λ3 that belongs to the type III IFN-λ family. Type I and III IFNs induce transcription of IFN-stimulated genes (ISGs), which mediate their antiviral effects [20, 21]. There was an association between IL28B genotype and intrahepatic expression levels of ISGs [22, 23]. IL28B genotypes also affect the intrahepatic innate immune system such as Toll-like receptor 3 and retinoic acid-inducible gene I signaling pathways [24, 25].

The favorable IL28B genotype is associated with induction of ISGs in liver and PBMCs after the initial administration of PEG-IFN/RBV in HCV patients and therefore accompanies with sustained virus response [24, 25]. 

Human dendritic cells (DCs) recognize HCV and produce IFN-λs [26, 27]. Patients with a favorable IL28B genotype produce more IFN-λ3. On the other hand, IFN-λ production from dendritic cells is significantly increased due to IFN-α stimulation [27]. Therefore, during exogenous IFN-α therapy, IFN-λ production by DCs is significantly increased and this pathway can explain how IL28B genetic variants predict the response to IFN-α therapy [27].

HCV is a major cause of end-stage liver disease worldwide and the patients frequently undergo liver transplantation, but recurrent hepatitis C in transplanted organ is still a major cause of morbidity [28].

In a study conducted on HCV-positive liver transplant recipients and donors, the IL28B rs8099917 was significantly associated with a sustained virus response after PEG-IFN/RBV therapy. Intrahepatic expression of IL28 messenger RNA was lower in donors and recipients that carried the minor alleles (T/G or T/T) [29].

Bitetto and colleagues investigated the possible association between recipient IL-28B rs12979860 and the occurrence of ACR after liver transplantation. They found that rejection is more common with TT genotype. The rejection happens more frequently from HCV-negative recipients carrying the C/C genotype compared to HCV-negative patients with at least one T allele. In HCV-positive patients, recipients with at least one C allele experience more rejection episodes than patients carrying the T/T genotype [16]. In our study, no association was found between IL-28B rs12979860 polymorphism and acute rejection in transplant recipients. 

In patients with cirrhosis who underwent transplantation, T allele was more prevalent in HCV-positive patients in comparison to non-HCV patients. Furthermore, HCV-positive recipients with rs12979860 IL28B T/T genotype had the highest frequency of acute rejection episodes [30]. Each rejection episode accompanied with inflammation and accelerated the fibrosis progression in liver allograft [31]. Overall, the inheritance of T allele is accompanied with more complication after transplantation [30].

As HCV was not the major cause of cirrhosis in our population, the effect of IL28B genotype on HCV recurrence or response to treatment after transplantation could not be evaluated in our study. The major finding of the current study was that patients with inheritance of **C** allele variant were at increased risk of liver fibrosis and cirrhosis. In respect of IL-28B polymorphism, as a predisposing factor for cirrhosis, controversial reports are mentioned in the literature.

Barreiro, *et. al.*, analyzed the association of IL28B genotype with risk of cirrhosis in HIV/HCV co-infected patients. They revealed that favorable IL28B CC genotype is associated with more liver inflammation and fibrosis [32]. In a cohort study conducted by Noureddin, *et. al.*, patients with CC genotype at rs12979860 had significantly higher portal inflammation and ALT levels at initial liver biopsy. However, in follow-up biopsy, no difference in fibrotic progression was identified between CC and non-CC genotypes [33].

Marabita, *et. al.*, found no association between IL28B genotype and progression of fibrosis [34].

In conclusion, no association was found between IL-28B rs12979860 polymorphism and acute rejection in transplant recipients. However, inheritance of **C** allele variant may change the intrahepatic expression levels of ISGs and thus it may be considered a risk factor for progression of liver damage toward cirrhosis.
